# Estimate and needs of the transgender adult population: the SPoT study

**DOI:** 10.1007/s40618-023-02251-9

**Published:** 2024-02-19

**Authors:** A. D. Fisher, M. Marconi, G. Castellini, J. D. Safer, S. D’Arienzo, M. Levi, L. Brogonzoli, R. Iardino, C. Cocchetti, A. Romani, F. Mazzoli, P. Matarrese, V. Ricca, L. Vignozzi, M. Maggi, M. Pierdominici, J. Ristori

**Affiliations:** 1 Andrology, Women’s Endocrinology and Gender Incongruence Unit, Florence University Hospital, University of Florence, Florence, Italy; 2 Reference Centre for Gender Medicine, Italian National Institute of Health, Rome, Italy; 3 Psychiatric Unit, University of Florence, Florence, Italy; 4 Mount Sinai Center for Transgender Medicine and Surgery, New York City, NY USA; 5 Icahn School of Medicine at Mount Sinai, New York City, NY USA; 6 Azienda USL Toscana Centro SOC Monitoraggio e Programmazione Performance Clinico-Assistenziale Pistoia, Prato ed Empoli e Relazioni con Agenzie Esterne, Florence, Italy; 7 UFC Epidemiologia, Dipartimento di Prevenzione Azienda USL Toscana Centro, Florence, Italy; 8 Fondazione The Bridge, Milan, Italy; 9 Department of Experimental, Clinical and Biomedical Sciences, University of Florence, Florence, Italy; 10 Andrology, Women’s Endocrinology and Gender Incongruence Unit, Careggi University Hospital, University of Florence, Viale Pieraccini 6, 50139 Florence, Italy

**Keywords:** Estimates, Survey, Transgender population, Binary, Nonbinary

## Abstract

**Background:**

Despite the increasing interest in transgender health research, to date little is known about the size of the transgender and gender diverse (TGD) population.

**Methods:**

A web-based questionnaire survey was developed, including a collection of socio-demographic characteristics and disseminated online through social media. Gender incongruence was evaluated by using a 2-item approach assessing gender recorded at birth and gender identity. The primary objective of the present population-based study was to estimate the proportion of TGD people across ages among a large sample of people who answered a web-based survey. The secondary endpoints were to identify gender-affirming needs and possible barriers to healthcare access.

**Results:**

A total of 19,572 individuals participated in the survey, of whom 7.7% reported a gender identity different from the sex recorded at birth. A significantly higher proportion of TGD people was observed in the youngest group of participants compared with older ones. Among TGD people who participated in the study, 58.4% were nonbinary, and 49.1% experienced discrimination in accessing health care services. Nonbinary TGD participants reported both the need for legal name and gender change, along with hormonal and surgical interventions less frequently compared to binary persons.

**Conclusions:**

Being TGD is not a marginal condition In Italy. A large proportion of TGD persons may not need medical and surgical treatments. TGD people often experience barriers to healthcare access relating to gender identity.

**Supplementary Information:**

The online version contains supplementary material available at 10.1007/s40618-023-02251-9.

## Introduction

Transgender and gender diverse (TGD) people represent a broad spectrum of individuals whose gender identities differ from the recorded sex at birth [1, 2]. Despite the increasing interest in transgender health research, to date little is known about the real size of the TGD population, mostly due to the heterogeneity of this population, and the lack of information regarding gender identity in health record systems [2, 3].

Currently, most information comes from clinical-based studies, involving TGD people seeking gender-affirming hormonal treatments [4–9]. This approach might be associated with underestimation of the real proportion of TGD people. Some TGD people start hormonal treatment without medical supervision and others do not seek any gender-affirming treatment [10–12]. Furthermore, referral to knowledgeable providers may be hindered by perceived stigma, marginalization, social and financial constraints, and lack of knowledgeable providers [10, 13, 14].

The limited number of available population-based studies report estimates of TGD population size [15] ranging from 0.3 to 4.5% among adults [16]. Moreover, studies estimating TGD population size should employ a two-step method, involving the universal query of both gender identity and sex recorded at birth [16, 17].

To date, the demographics of the TGD population in Italy have not been characterized. One study hypothesized a proportion of 0.9 per 100,000 based on the number of gender-affirming surgeries [6]. Accurate estimates of the size, composition and needs of the TGD population are essential to plan appropriate healthcare services.

To bridge this gap, the SPoT study (*Stima della popolazione transgender adulta in Italia,* “Estimate of the transgender adult population in Italy”) was promoted by the Careggi University Hospital—University of Florence, in collaboration with the National Institute of Health in Italy (ISS) and The Bridge Foundation, and with the support of the Italian National Observatory on Gender Identity (ONIG). The main aims of the present population-based study were to begin to assess the size of the adult TGD population starting with a large sample of Italian people who answered a two-step method online questionnaire and to query for gender-affirming needs.

## Methods

### Study design and population

An ad hoc web-based questionnaire survey (Google Forms) was developed and disseminated online through radio channels as well as social media i.e., Facebook, Instagram. The aim was to reach a large sample of the population. On the basis of data from the international literature regarding the size of the TGD population [15, 16], we hypothesised that 1 ± 0.5% of the Italian adult population would be TGD. Thus, we aimed to enroll 7,610 study participants. The study started in December 2019 and closed in December 2021 (total duration: 24 months).

The inclusion criteria for the study were individuals aged 18 years and over and residing in Italy. Participation in the survey was voluntary. The questionnaire took less than 3 min to complete and contained 13 closed-ended questions (Appendix A). Questions one to seven, open to the entire population, were designed to gather information on the participants’ sociodemographic characteristics. To determine TGD population size and to capture the range of TGD people with nonbinary gender identities, a two-step method was used (with gender identity choices ‘‘man’’, ‘‘woman’’, ‘‘[neither] man, [nor] woman’, “other”). TGD individuals—those whose gender identity differed from their sex recorded at birth—were asked to respond to additional questions. The additional questions were aimed to define specific health needs, age of gender incongruence awareness, the wish to undergo a social and/or medical gender-affirming path, and to identify experienced inequalities in accessing healthcare services because of their gender identity.

Whenever appropriate, a four-point ordinal scale was used: “always”, “often”, “sometimes” and “never”. The questionnaire translated to English is available in the Supplementary Appendix.

Ethical approval for this study was obtained from the institutional review board at the University of Florence, Research Ethical Committee (Prot. N. 25 June 25, 2019). Informed consent was waived, given that data collection was anonymized.

### Primary and secondary endpoints

The primary objective was to assess the proportion of TGD population among adults (i.e., older than 18 years) in Italy who participated in a large web-based survey. The secondary endpoint was to assess the needs of TGD people who answered the questionnaire including legal name and gender marker change, hormonal and/or surgical gender-affirming treatment needs, and to report barriers to healthcare access related to gender identity.

### Statistical analysis

Proportion with the respective 95% confidence interval (CI) was calculated for each of the following groups: cis- and transgender, masculine spectrum and feminine spectrum, and nonbinary. TGD people were defined as those reporting any gender identity different from the sex recorded at birth, while cisgender as those having a gender identity matching their sex recorded at birth. Among TGD people, binary was defined as those with a gender identity opposite of the sex recorded at birth while nonbinary was defined as those reporting any other gender identity. Frequency measures and contingency tables were used to summarize and analyse the relationships with categorical variables. Sociodemographic characteristics were compared across groups. To explore the association with key study variables, the independent sample t-test and Chi-squared test were used, to evaluate the associations with, respectively, continuous (i.e., age) and categorical variables. Logistic regression was used for multivariate analyses, adjusting for age, whenever appropriate. All analyses were performed using STATA software, version 15 (STATA Corp, College Station, TX).

## Results

A total of 19,572 individuals participated in the survey. A point proportion of 7.7% (95% C.I. 7.3–8.1) was observed for TGD status, as 1,501 declared a gender identity different from the sex recorded at birth. Among participants TGD people were significantly younger than cisgender ones (median age 26[19; 83] vs. 36 years [19; 83]; *p* < 0.001). Socio-demographic characteristics of the study participants are reported in Table 1 and their significant differences in Supplemental Table 1B. Results were confirmed after adjusting for age (data not shown). When the sample was stratified in tertiles according to age (18–29, 30–39 and ≥ 40, years old, respectively), a significantly higher proportion of TGD people was observed in the youngest group of participants compared to the mid and older ones (14.7%, 4.1% and 4.8%, in each tertile, *p* < 0.001).

**Table 1:** Socio-demographic characteristics of the total sample (question one to seven), stratified according to gender identity and sex recorded at birth

	Cisgender people		TGD people														Total	
			Total		Binary people						Nonbinary people							
					Total		Birth recorded females		Birth recorded males		Total		Birth recorded females		Birth recorded males			
*N*	18,071		1501		624		452		172		877		619		258		19,572	
%	92.3%		7.7%		41.6%		72.4%		27.6%		58.4%		70.6%		29.40%		100%	
Age (*,^§,°,#^)																		
MEDIAN	36		26		25		23		33		27		25		39		35	
[MIN; MAX]	[19; 83]		[19; 83]		[19; 73]		[19; 67]		[19; 73]		[19; 83]		[19; 70]		[19; 83]		[19; 83]	
P25	28		22		21		21		24		22		21		28		27	
P75	43		38		34		29		47		39		33		48		43	
IQR	15		16		13		8		23		17		12		20		16	

Nationality (*^,&^)	*N*	%	*N*	%	*N*	%	*N*	%	*N*	%	*N*	%	*N*	%	*N*	%	*N*	%
Italian	17,862	98.8	1464	97.5	608	97.4	445	98.5	163	94.8	856	97.6	607	98.1	249	96.5	19,326	98.7
Not Italian	209	1.2	37	2.5	16	2.6	7	1.5	9	5.2	21	2.4	12	1.9	9	3.5	246	1.3

Geographic distribution (*,^§,#^)	*N*	%	*N*	%	*N*	%	*N*	%	*N*	%	*N*	%	*N*	%	*N*	%	*N*	%
Northern Italy	12,698	70.3	886	59.0	340	54.5	245	54.2	95	55.2	546	62.3	381	61.6	165	64.0	13,584	69.4
Central Italy	3727	20.6	325	21.7	132	21.2	89	19.7	43	25.0	193	22.0	138	22.3	55	2103	4052	20.7
Southern Italy	1099	6.1	184	12.3	97	15.5	79	17.5	18	10.5	87	9.9	59	9.5	28	10.9	1283	6,.6
Islands	547	3.0	106	7.1	55	8.8	39	8.6	16	9.3	51	5.8	41	6.6	10	3.9	653	3.3

Size of municipality of residence (*,^@^)	*N*	%	*N*	%	*N*	%	*N*	%	*N*	%	*N*	%	*N*	%	*N*	%	*N*	%
Large municipality (> 250.000 inhabitants)	3818	21.1	464	30.9	194	31.1	129	28.5	65	37.8	270	30.8	184	29.7	86	33.3	4282	21.9
Middle municipality (5.000–250.000 inhabitants)	10,941	60.5	826	55.0	350	56.1	259	57.3	91	52.9	476	54.3	342	55.3	134	51.9	11,767	60.1
Small municipality (< 5.000 inhabitants)	312	18.3	211	14.1	80	12.8	64	14.2	16	9.3	131	14.9	93	15.0	38	14.7	3523	18.0

Educational level (*,^§,#,@^)	*N*	%	*N*	%	*N*	%	*N*	%	*N*	%	*N*	%	*N*	%	*N*	%	*N*	%
University degree	7588	42.0	455	30.3	145	23.2	94	20.8	51	29.7	310	35.3	208	33.6	102	39.5	8043	41.1
High school degree	9228	51.1	904	60.2	410	65.7	311	68.8	99	57.6	494	56.3	369	59.6	125	48.4	10,132	51.8
Secondary school degree	1207	6.7	134	8.9	67	10.7	47	10.4	20	11.6	67	7.6	39	6.3	28	10.9	1341	6.9
None/primary school degree	48	0.3	8	0.5	2	0.3	0	0.0	2	1.2	6	0.7	3	0.5	3	1.2	56	0.3

Data are expressed as percentages and absolute number

The level of significance is expressed as *p* values

*p < 0.001. Differences between cisgender and TGD people

^§^*p* < 0.001. Differences between binary and nonbinary people

^°^*p* < 0.001. Differences between binary and nonbinary people among birth recorded males TGD sample

^#^*p* < 0.001. Differences between binary and nonbinary people among birth recorded females TGD sample

^&^*p* < 0.005. Differences between birth-recorded females and birth-recorded males people among binary TGD sample

^@^*p* < 0.01 Differences between birth-recorded females and birth-recorded males people among nonbinary TGD sample

**Fig. 1:**
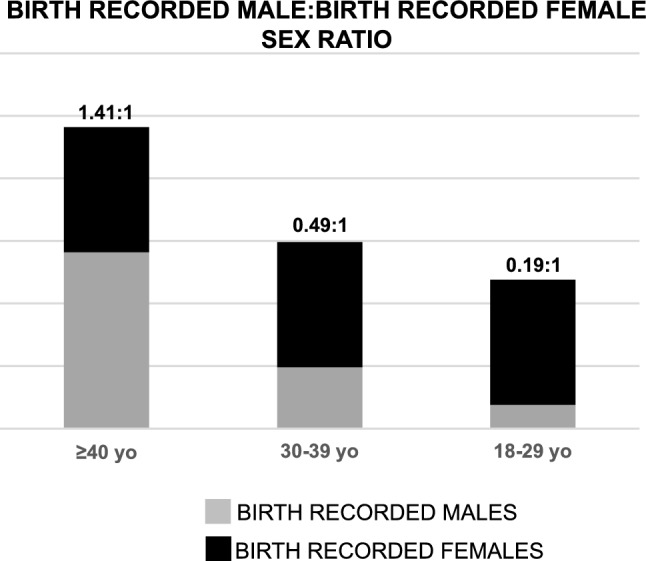
Birth recorded males: birth recorded females sex ratio of TGD respondents across age tertiles

Among TGD people, 58.4% (95% C.I. 55.9–60.9) were nonbinary, and 41.6% (95% C.I. 39.0—44.1) were binary, with a significant increase in the nonbinary proportion in the youngest tertile *vs*. the mid and older ones (7.9% vs. 2.8% and 3.1%, respectively for the youngest, mid and oldest tertiles, *p* < 0.001). Birth-recorded females participated in the study more than birth-recorded males in the younger 2 age tertiles (73% female at birth vs. 27% male at birth, and 67% female at birth vs. 33% male at birth, respectively), while birth-recorded males were more represented in the oldest tertile (42% female at birth vs. 58% male at birth). Figure 1 shows the birth recorded sex ratio across age tertiles. The temporal trend among age in shifting birth recorded ratio was confirmed also when binary and nonbinary participants subsamples were considered (birth recorded male:birth recorded female 0.21:1; 0.55:1; 1.33:1 for binary TGD people and 0.18:1, 0.46:1, 1.45:1, for nonbinary people). Nonbinary TGD participants were more likely (*p* < 0.001) to reside in the regions of Northern Italy than binary TGD participants (62.3% vs. 54.5%), whereas binary ones were more likely to live in Southern Italy (15.5% vs. 9.9%) and in the islands (Sicily and Sardinia; 8.8% vs. 5.8%). Nonbinary TGD participants had a statistically significant higher education level (*p* < 0.001) than binary participants. No statistically significant differences between binary and nonbinary TGD participants were observed concerning nationality and the size of the municipality of residence. Among binary TGD people, birth-recorded females were more likely to be based in municipalities with less than 5,000 inhabitants than birth-recorded males (*p* = 0.041), who were more represented than birth-recorded females in municipalities with > 250,000 inhabitants.

Regarding the specific questions targeting gender incongruence experience (Table 2 and Supplemental Table 2B), nonbinary participants had gender identity awareness later in life than binary participants (also when stratified according to sex recorded at birth). Almost double the binary sample, compared to the nonbinary one, reported gender incongruence awareness during childhood. In the case of nonbinary TGD people, no statistically significant difference was observed regarding when self-reported gender incongruence began. Among binary people, birth-recorded females reported gender incongruence awareness during childhood more frequently when compared to birth-recorded males (*p* < 0.001).

**Table 2:** Reported answers for questions eight to 13 for TGD individuals, stratified according to gender identity and sex recorded at birth

	TGD people													
	Total		Binary						Nonbinary					
			Total		Birth recorded females TGD		Birth recorded males TGD		Total		Birth recorded females TGD		Birth recorded males TGD	
*N*	1501		624		452		172		877		619		258	

Gender incongruence awareness (^§,&,°,#^)	*N*	%	*N*	%	*N*	%	*N*	%	*N*	%	*N*	%	*N*	%
Childhood	539	35.9	309	49.5	230	50.9	79	45.9	230	26.2	157	25.4	73	28.3
Early puberty (8–13 yo recorded females; 9–14 yo recorded males)	360	24.0	151	24.2	94	20.8	57	331	209	23.8	134	21.6	75	29.1
Late puberty (14–18 yo recorded females; 15–18 yo recorded males)	306	20.4	104	16.7	87	19.2	17	9,9	202	23.0	161	26.0	41	15.9
Adulthood	296	19.7	60	9.6	41	9.1	19	11,0	236	26.9	167	27.0	69	26.7

Desire for body changes (^§,&,@,°,#^)	*N*	%	*N*	%	*N*	%	*N*	%	*N*	%	*N*	%	*N*	%
Never	297	19.8	18	2.9	8	1.8	10	5,8	279	31.8	170	27.5	109	42.2
Sometimes	354	23.6	56	9.0	31	6.9	25	14.5	298	34.0	223	36.0	75	29.1
Often	272	18.1	81	13.0	60	13.3	21	12.2	191	21.8	143	23.1	48	18.6
Always	578	38.5	469	75.2	353	78.1	116	67.4	109	12.4	83	13.4	26	10.1

Desire to legally change name/gender (^§,&,@,°,#^)	*N*	%	*N*	%	*N*	%	*N*	%	*N*	%	*N*	%	*N*	%
Yes	842	56.1	584	93.6	432	95.6	152	88.4	258	29.4	198	32.0	60	23.3
No	659	43.9	40	6.4	20	44	20	11.6	619	70.6	421	68.0	198	76.7

Desire for gender affirming hormonal treatment, GAHT (^§,°,#^)	*N*	%	*N*	%	*N*	%	*N*	%	*N*	%	*N*	%	*N*	%
Never	564	37.6	30	4.8	22	4.9	8	4.7	534	60.9	378	61.1	156	60.5
Yes, I haven’t done it yet, I’m about to start it	541	36.0	255	40.9	196	43.4	59	34.3	286	32.6	214	34.6	72	27.9
Yes, I'm currently on GAHT	364	24.3	325	52.1	231	51.1	94	54.7	39	4.4	17	2.7	22	8.5
Yes, I’ve done in the past it but I’m currently not doing it	32	2.1	14	2.2	3	0.7	11	6.4	18	2.1	10	1.6	8	3.1

Desire for gender-affirming surgery (^§,&,@,°,#^)	*N*	%	*N*	%	*N*	%	*N*	%	*N*	%	*N*	%	*N*	%
No	551	36.7	44	7	15	3.3	29	16.9	507	57.8	338	54.6	169	65.5
Yes, I've already done it	128	8.5	106	17.0	69	15.3	37	21.5	22	2.5	9	1.5	13	5.0
Yes, I havent' done it yet, I have planned to do it	822	54.8	474	76	368	81.4	29	61.6	348	39,7	272	43.9	76	29.5

Perceived discrimination (^§,&,^ *^, #^)	*N*	%	*N*	%	*N*	%	*N*	%	*N*	%	*N*	%	*N*	%
No, never	606	40.4	183	29.3	112	24.8	71	41.3	423	48.2	281	45.4	142	55.0
Yes, always/sometimes	585	39.0	337	54.0	268	59.3	69	40.1	248	28.3	177	28.6	71	27.5
I never accessed healthcare services	310	20.7	104	16.7	72	15.9	32	18.6	206	23.5	161	26.0	45	17.4

Perceived discrimination in people who accessed haealth care services (*N* = 1191) (^§,&,^ *^, #^)	*N*	%	*N*	%	*N*	%	*N*	%	*N*	%	*N*	%	*N*	%
No, never	606	50.9	419	35.2	351	29.5	833	50.7	750	63.0	731	61,4	794	66.7
Yes, always/sometimes	585	49.1	772	64.8	840	70.5	587	49.3	441	37.0	460	38.6	397	33.3

Data are expressed as percentages and absolute number

^§^*p* < 0.001. Differences between binary and nonbinary people

^&^*p* < 0.02. Differences between birth-recorded females and birth-recorded males people among binary TGD sample

^@^*p* < 0.05 Differences between birth-recorded females and birth-recorded males people among nonbinary TGD sample

^°^*p* < 0.01. Differences between binary and nonbinary people among birth recorded males TGD sample

^#^*p* < 0.01. Differences between binary and nonbinary people among birth recorded females TGD sample

While most binary TGD participants (75.2%) declared a persistent need in the previous six months to make external anatomy more congruent with gender identity, almost two-thirds of nonbinary participants (65.8%) reported that they never or only sometimes had felt such necessity. While almost all (93.6%) binary TGD people who answered the questionnaire had felt the need to legally change their name and gender marker at some point, over two-thirds (70.6%) of nonbinary TGD participants never felt such necessity (*p* < 0.001). When participants were stratified according to the sex recorded at birth, in both binary and nonbinary subsamples of birth-recorded female TGD people more often reported the wish to change their body as well as to legally change name and gender marker compared to birth-recorded male participants (both *p* < 0.05).

While the great majority of binary TGD participants (95.2%; birth recorded female: 95.1%; birth recorded male: 95.3%) had past, present, or future planned gender-affirming hormone treatment, over 60% of nonbinary TGD declared no such use (*p* < 0.001). No statistically significant difference was observed among birth-recorded females and birth-recorded males.

More than half (54.6%) of nonbinary TGD people who participated in the study had never felt the need for gender-affirming surgery, while the majority of binary TGD people (92.9%, *p* < 0.001) reported such a need. Binary people declared more often they had already undergone gender-affirming surgery at the time of the survey compared to nonbinary ones (17% vs. 2.5%, respectively, *p* < 0.001), whereas 57.8% of nonbinary people (birth recorded female: 54.6%; birth recorded male: 65.5%) who were interested in surgery had not had surgery at the time of the survey.

The 20.7% of the participants reported they had never accessed healthcare services. Of those, rates were higher among nonbinary relative to binary participants (23.5% vs. 16.7%, *p* < 0.001). Among TGD people who had contact with healthcare services (N = 1,191), half (49.1%) had always or sometimes felt barriers accessing healthcare services because of their gender identity. This was more often observed for binary (64.8%) versus nonbinary (37.0%) TGD people (*p* < 0.001), even more for binary birth recorded females (70.5%, *p* < 0.001).

## Discussion

This is the first population-based study to attempt to assess TGD population estimates and needs in Italy. Compared to clinic-based studies (which are typically limited to individuals seeking treatment) this survey included a broader and more inclusive population [3].

As expected, the proportion of TGD people in the population was found to be higher than statistics based on health system data (ranging from 0.02 to 0.03%; [18, 19]), but also as compared with other internet-based surveys (in two studies from the Netherlands 1.1% and 4.6%, respectively, for birth recorded males and 0.8–3.2%, for birth recorded females; in Belgium, 0.7% for birth recorded males and 0.6% for birth recorded females; in two studies from Sweden, 2.3% and 2.8%, respectively, of the total sample; [20–22].

The only estimate comparable to the one obtained in the present study was a survey [23] derived from a school-based sample in Florida and California, which found the proportion of TGD participants to be 8.4% of the sample. We can speculate that this study provided a similar selection of the sample, adopting a broader definition of gender diversity. Furthermore, it’s possible that TGD people were more motivated to respond to the questionnaire, leading to an overestimation of the TGD respondents in comparison with the cisgender ones. However, these data could be a relevant insight into the real numbers of the TGD population in Italy.

One of the strengths of the present study is the use of the so-called two-step method [16, 17], which identified more nonbinary people, giving visibility to TGD persons who, otherwise, would not consider themselves as so. Indeed, to date, few studies have used this method, which could partially overcome the heterogeneity in the estimation of the numbers of the TGD community [16].

The increasing proportion of TGD people detected in more recent studies as well as in younger age groups might also result from socio-political advances including several innovations regarding transgender care in many national scenarios, less pronounced cultural stigma, and changes in referral patterns [16]. Also, easier access to information through the web may have helped gender identity awareness; il line, a previous study [24], reported an association between increasing TGD-related topics in the media and numbers of young people presenting to gender clinics.

The temporal trend among generations in decreasing birth-recorded male to birth-recorded female TGD ratio, from predominantly birth-recorded male trans people to predominantly birth-recorded female across age tertiles in the present population-based study, is in line with previous studies analysing referrals to clinics as well as data from integrated health systems [2, 16]. The change in this temporal trend is confirmed for the first time in a population-based study. The specific reasons of this phenomenon are far from being understood; however, it could be part of the so-called “generational effect”, defined as the variation in one population parameter according to the year of birth, often coinciding with other shifts in population characteristics in the same time [16]. The TGD population reported a lower level of education relative to the age-adjusted cisgender population. This result might be interpreted as a further demonstration of the difficulties that TGD people have in accessing the education system. Young TGD people often face intolerance at home or school [10]. Thus, stigma and intolerance may be considered explanations in part for reduced educational level in TGD people.

Several TGD persons reported difficulty changing their gender marker at school or university [10, 13]. Indeed, TGD persons’ identity documentation is often incongruent with their gender identity and thus reveals their being transgender. A National Dutch Survey reported that 42% of TGD persons received negative reactions because of their transgender identity, most commonly in public (38%) and at school (21%) [25]. A survey involving 6,450 TGD people in the United States reported that 15% of TGD students dropped out of school as the result of perceived and/or internalized stigma [26]. Perceived stigma was found also in health care services. A large proportion of TGD people report discrimination and problems in accessing health care. In general, it has been documented that LGBTQ individuals report inadequate care due to previous stigmatizing experiences in healthcare settings [27, 28].

The present study highlights the importance of considering the heterogeneity of the TGD population, as the majority of the TGD group was composed of nonbinary persons. The term nonbinary is used to include a broader range of persons as compared to previous investigations [2]. It is important to note that not all nonbinary people consider themselves to be transgender. Indeed, many persons consider the label TGD only within the gender binary with a report that some do not feel “trans enough” to describe themselves as transgender [2].

In the present study, TGD people who self-identified as nonbinary had a number of different characteristics relative to those TGD people identified as binary, including geographical area, awareness of gender identity as well as need for gender-affirming treatment.

For example, the greater proportion of nonbinary persons in the Northern vs. Southern regions of Italy might be interpreted in light of the association between the reduced need for dichotomous self-definitions and increased acceptance of gender variance in more tolerant and open-minded environmental contexts [22]. This interpretation is also supported by the higher education level of nonbinary persons. Regarding geographical patterns, few studies evaluated this information, potentially biased by socio-cultural differences across countries. For example, we found that birth recorded female persons tend to live more in small centres, while Crissman et al. [29] found birth recorded male persons were more likely to live in rural areas in the US. Furthermore, an effect of age on gender identity awareness was associated with binary vs. nonbinary people. Confirming previous observations [30, 31], most of the binary persons reported a discrepancy between recorded sex and gender identity before puberty, while no effect of age was observed for nonbinary persons.

Finally, as previously reported, the majority of binary TGD persons requested medical gender-affirming treatment, such as hormones and/or surgeries. This result is consistent with previous findings in clinical and non-clinical populations [32–35]. Nonbinary persons reported less categorical (and maybe less stereotyped) needs, with fewer persons asking for legal changes or medical interventions.

The survey showed that a large proportion of TGD persons (especially those with a nonbinary gender identity) may not need medical or surgical intervention. Different life trajectories may be described, especially on the basis of being more binary *vs*. nonbinary.

In conclusion, being TGD is clearly not a marginal, rare condition. A large proportion of TGD persons (especially binary ones) need medical and surgical treatment, which are not adequately provided currently in Italy (according to the services’ map published on the ISS website https://www.infotrans.it/en-home). As recommended by the WPATH SOC 8 [2], healthcare systems should provide medically necessary interventions for the health and well-being of TGD individuals. While a large number of TGD persons do not need hormonal or surgical interventions, consequences of stigma (such as education and access to health care) emerge as important concerns to address.

### Limitations

The results of the present study should be considered in light of some limitations. First, responses and rates should be evaluated in relation to selection bias. People attracted to the survey might have been more open-minded persons. TGD people may have been more likely to answer the questionnaire, a limit to the generalizability of the results. Another important weakness of the study was the lack of response rate information related to the study design (web-based questionnaire survey).

In addition, the interpretation of results of the present survey should take into consideration the demographic distribution of the sample, which was disproportionately composed of birth-recorded females and young people. This disproportion is partly due to the sampling method and the sources used for recruiting participants, such as social media and radio channels, which tend to have a higher engagement by individuals recorded at birth as female. However, this explanation alone does not fully explain the observed generational trend observed. Indeed, we have noted that the proportion of birth-recorded females is higher among younger generations within the TGD community. This suggests that there may be generational shifts contributing to this trend, possibly due to cultural changes, increased visibility and acceptance of TGD individuals. Further research is necessary to explore these dynamics in depth and understand their implications. Finally, considering the self-report nature of the survey, and the lack of in person assessment, it is not possible to identify mental health problems or other psychosocial concerns which could be self-misattributed to being TGD.

### Supplementary Information

Below is the link to the electronic supplementary material.

Supplementary file1 (DOCX 27 KB)

Supplementary file2 (DOCX 16 KB)

Supplementary file3 (DOCX 16 KB)
