# Supplementary material for: Estimate and needs of the transgender adult population: the SPoT study
Source: J Endocrinol Invest. 2024 Feb 19;47(6):1373–83. doi: 10.1007/s40618-023-02251-9 (PMC11143024; doi:10.1007/s40618-023-02251-9)
Supplement: Supplementary file 1 — Supplementary file1 (DOCX 27 KB) [file 40618_2023_2251_MOESM1_ESM.docx]

# **Appendix A. The web-based questionnaire**

1. **How old are you? □□**
2. **What is your Nationality?**

**□** Italian

**□** Not Italian, EU

**□** Not Italian, extra EU

**3) In which part of Italy do you live?**

□ Islands

□ Southern

□ Center

□ Northern

**4) Please indicate the number of residents in your municipality**

□ > 250,000

□ between 5,000 and to 250,000

**□** < 5,000

1. **What is the highest degree or level of education you have completed?**

**□** None/Primary School
**□** Middle school
**□** High school

**□** Bachelor's degree/ Master’s Degree

1. **What was your sex recorded at birth?**

**□** Male

**□** Female

**7) To which gender identity do you most identify?**

□ Man

□ Woman

□ Both man and woman

□ Not man nor woman

□ Other (please specify)

**End of questionnaire**

**Thank you for participating in our survey**

**Based on the answer given to questions 6 and 7, the questions of the sections for birth recorded females or for birth recorded males TGD people will open**

**Section for birth recorded females TGD people**

Aims of the research

The following questions aim to define the needs related to trans-specific health care and to lay the foundations for a broader recognition of the health needs of the transgender population

**8) At what age did you begin to perceive a gender identity other than the sex recorded at birth?**

□ before the age of 7

□ between 8 and 13 years old

□ between 14 and 18 years old

□ after the age of 18

**9) In the past 6 months, have you ever wanted to change your body to make it more like how you feel?**

□ Always

□ Often

□ Sometimes

□ Never

**10) Have you ever wanted to change your legal name and gender?**

**□** No

□ Yes, but I have not yet started the legal process

□ Yes, and I have started the legal process

□ Yes, I have already changed my legal name and gender

**11)** **Have you ever wanted a hormonal treatment to make you more masculine and/or to modify your body in order to make it more in line with your gender identity?**

**□** No

□ Yes, but I haven't done it yet

□ Yes, I'm about to start it

□ Yes, I am currently taking hormones

□ Yes, I've taken hormones in the past but not now

**12) Have you ever wanted to undergo surgery to make your body more masculine and/or to modify your body to make it more in line with your gender identity?**

□ No

□ Yes, but I haven't done it yet

□ Yes, I have already programmed to do it

□ in Italy in the public sector

□ in Italy in the private sector

□ abroad with reimbursement from the National Health Service

□ abroad in the private sector

□ Yes, I already have had surgery

□ in Italy in the public sector

□ in Italy in the private sector

□ abroad with reimbursement from the Italian National Health Service

□ abroad in the private sector

**13) Have you ever felt discriminated and/or have you ever encountered problems in accessing or using health services based on your gender identity and/or expression?**

**□** Yes, always

□ Yes, sometimes

□ No, never

□ I have never turned to health services

**End of questionnaire**

**Thank you for participating in our survey**

**Section for birth recorded**

**males TGD people**

Aims of the research

The following questions aim to define the needs related to trans-specific health care and to lay the foundations for a broader recognition of the health needs of the transgender population

**8) At what age did you begin to perceive a gender identity other than the sex recorded at birth?**

□ before the age of 8

□ between 9 and 14 years old

□ between 15 and 18 years old

□ after the age of 18

**9) In the past 6 months, have you ever wanted to change your body to make it more like how you feel?**

□ Always

□ Often

□ Sometimes

□ Never

**10) Have you ever wanted to change your legal name and gender?**

**□** No

□ Yes, but I have not yet started the legal process

□ Yes, and I have started the legal process

□ Yes, I have already changed my legal name and gender

**11)** **Have you ever wanted a hormone treatment to make you more feminine and/or to modify your body in order to make it more in line with your gender identity?**

**□** No

□ Yes, but I haven't done it yet

□ Yes, I'm about to start it

□ Yes, I am currently taking hormones

□ Yes, I've taken hormones in the past but not now

**12) Have you ever wanted to have surgery to make your body more feminine and/or to modify your body to make it more compliant with your gender identity?**

□ No

□ Yes, but I haven't done it yet

□ Yes, I have already programmed to do it

□ in Italy in the public sector

□ in Italy in the private sector

□ abroad with reimbursement from the National Health Service

□ abroad in the private sector

□ Yes, I already have had surgery

□ in Italy in the public sector

□ in Italy in the private sector

□ abroad with reimbursement from the Italian National Health Service

□ abroad in the private sector

**13) Have you ever felt discriminated and/or have you ever encountered problems in accessing or using health services based on your gender identity and/or expression?**

**□** Yes, always

□ Yes, sometimes

□ No, never

□ I have never turned to health services

**End of questionnaire**

**Thank you for participating in our survey**
