# Supplementary material for: Estimate and needs of the transgender adult population: the SPoT study
Source: J Endocrinol Invest. 2024 Feb 19;47(6):1373–83. doi: 10.1007/s40618-023-02251-9 (PMC11143024; doi:10.1007/s40618-023-02251-9)
Supplement: Supplementary file 2 — Supplementary file2 (DOCX 16 KB) [file 40618_2023_2251_MOESM2_ESM.docx]

**Supplemental Table 1B**. Levels of significance for the differences reported in Table 1.

|  | ***CISGENDER vs. TGD PEOPLE (*)*** | ***BINARY vs. NONBINARY TGD PEOPLE (§)*** | ***BIRTH RECORDED MALES TGD*** | ***BIRTH RECORDED FEMALES TGD*** | ***BINARY TGD*** | ***NONBINARY TGD*** |  |
| --- | --- | --- | --- | --- | --- | --- | --- |
|  |  |  | ***BINARY VS. NONBINARY TGD PEOPLE (°)*** | ***BINARY VS. NONBINARY TGD PEOPLE (#)*** | ***BIRTH RECORDED FEMALES VS. BIRTH RECORDED MALE TGD PEOPLE (&)*** | ***BIRTH RECORDED FEMALES VS. BIRTH RECORDED MALE TGD PEOPLE (@)*** |  |
| Age | **p<0.001** | **p<0.001** | **p<0.001** | **p<0.001** | **p<0.001** | p=0.7 |  |
| Nationality | **p<0.001** | p=0.835 | p=0.376 | p=0.633 | **p=0.009** | p=0.428 |  |
| Geographic distribution | **p<0.001** | **p<0.001** | p=0.073 | **p=0.001** | p=0.128 | p=0.402 |  |
| Size of municipality of residence | **p<0.001** | p=0.499 | p=0.221 | p=0.797 | **p=0.041** | **p<0.001** |  |
| Educational level | **p<0.001** | **p<0.001** | p=0.209 | **p<0.001** | **p=0.007** | **p=0.007** |  |

|  |  |  |  |  |  |
| --- | --- | --- | --- | --- | --- |
