# Supplementary material for: Estimate and needs of the transgender adult population: the SPoT study
Source: J Endocrinol Invest. 2024 Feb 19;47(6):1373–83. doi: 10.1007/s40618-023-02251-9 (PMC11143024; doi:10.1007/s40618-023-02251-9)
Supplement: Supplementary file 3 — Supplementary file3 (DOCX 16 KB) [file 40618_2023_2251_MOESM3_ESM.docx]

**Supplemental Table 2B.** Levels of significance for the differences reported in Table 2.

|  | ***BINARY VS. NONBINARY TGD PEOPLE (§)*** | ***BINARY TGD PEOPLE*** | ***NONBINARY TGD PEOPLE*** | ***RECORDED MALES AT BIRTH*** | ***RECORDED FEMALES AT BIRTH*** |  |
| --- | --- | --- | --- | --- | --- | --- |
|  |  | ***RECORDED FEMALES AT BIRTH vs. RECORDED MALES AT BIRTH (&)*** | ***RECORDED FEMALES AT BIRTH vs. RECORDED MALES AT BIRTH (@)*** | ***BINARY VS. NONBINARY (°)*** | ***BINARY VS. NONBINARY (#)*** |  |
| Gender identity awareness | **p<0.001** | **p=0.001** | p=0.872 | **p<0.001** | **p<0.001** |  |
| Desire for body changes | **p<0.001** | **p=0.007** | **p=0.036** | **p<0.001** | **p<0.001** |  |
| Desire to legally change name/ gender | **p<0.001** | **p=0.001** | **p=0.01** | **p<0.001** | **p<0.001** |  |
| Desire for gender-affirming hormonal reatment | **p<0.001** | p=0.910 | p=0.051 | **p<0.001** | **p<0.001** |  |
| Desire for gender-affirming surgery | **p<0.001** | **p<0.001** | **p=0.039** | **p<0.001** | **p<0.001** |  |
| Perceived discrimination | **p<0.001** | **p<0.001** | p=0.425 | **p=0.003** | **p=0.003** |  |
